# Single enantiomer propeller-shaped polynuclear complexes as catalysts-proof-of-concept for enantioinduction in a Michael addition reaction

**DOI:** 10.1098/rsos.241537

**Published:** 2025-03-27

**Authors:** Joe Maddocks, Mohan Mahesh, Stavroula I. Sampani, Alexander C. Dixon, Christian D.-T. Nielsen, Prashant Kumar, Geoffrey R. Akien, John Spencer, Alaa Abdul-Sada, John F. C. Turner, Alan C. Spivey, George E. Kostakis

**Affiliations:** ^1^ Department of Chemistry, University of Sussex, Brighton, UK; ^2^ Department of Chemistry, Imperial College London, London, UK; ^3^ Department of Chemistry, Lancaster University, Lancaster, UK

**Keywords:** enantioselective induction, propeller-shaped complexes, Michael addition reaction

## Abstract

We report a family of propeller-shaped polynuclear metal complexes whose overall chirality is dictated by a single stereogenic centre within their component amino alcohol-ligand. These topologically intriguing complexes are readily prepared in enantiomerically pure form and are shown here to catalyse the conjugate addition of barbituric acids and their derivatives to nitroalkenes, with a catalyst loading of 1 mol%. Although only low levels of enantioinduction are observed, control experiments indicate that the enantioselectivity is dictated by the overall topology of the complex and not governed by binding to the tetrametallic entity, heralding a potentially new mode of catalysis.

Recent years have witnessed an explosion of interest in well-defined molecular species with structures that can be determined to atomic resolution and have constant dimensions within a set tolerance. For polynuclear complexes (PCs), interest in this field has focused on the relevance of these structures to bioinorganic chemistry and attempts to model the metal cluster at the heart of the oxygen-evolving centre of nature’s photosystem II [1,2]. Recently, PCs have also found applications as homogeneous catalysts [3,4]. However, the application of these entities as asymmetric catalysts is in its infancy, and the role of PCs in catalysis is not yet fully understood [5,6]. By contrast, traditional homogeneous metal-based catalysts, which consist of single metal atoms in combination with organic ligands to tune their activity and selectivity [7–9] have received extensive attention for decades, with their application and optimisation frequently assisted by computational analysis (e.g. using density functional theory (DFT)) [10]. However, their high nuclearity and sheer size make computational optimisation very time-consuming and expensive for PCs [11]. A further confounding factor when exploring catalysis involving PCs is that the bimetallic clusters are generally formed *in situ*, so the structures of the resultant active catalytic clusters are difficult to determine [12]. To develop a rational understanding of PCs in asymmetric catalysis, we considered an alternative approach. Herein we detail and implement a new synthetic approach that inspects topology in coordination chemistry for a higher-level description. This strategy is dependent upon single-crystal X-ray diffraction [13], Electron Paramagnetic Resonance (EPR) [14], Nuclear Magnetic Resonance (NMR) [15] and synthesis to permit correlation of structures in solution and solid form with their function as catalysts. This approach established the use of tetranuclear bimetallic 3d/4f PCs as Lewis acidic catalysts in a Michael addition reaction [16]. Fine-tuning the metal centres while retaining the topology of the catalyst allowed us to monitor the reaction with EPR, NMR and UV–Vis and identify the cooperative character of these bimetallic entities. To capitalize on these initial discoveries, we initiated a project to introduce chirality into this cooperative catalytic system. In this regard, we considered the chiral organic ligand H_2_L^R/S^ (figure 1).

**Figure 1.**
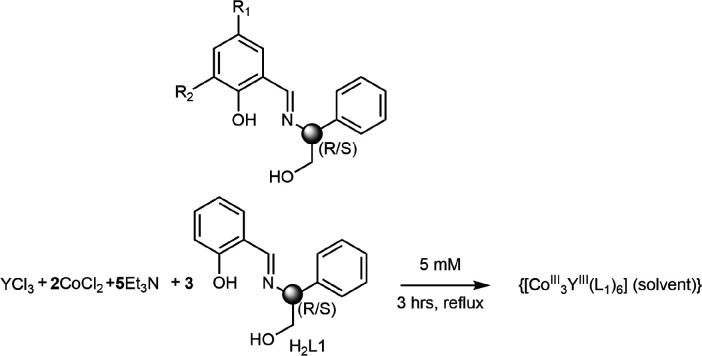
The organic ligands used in this study: H_2_ **L1** (R_1_ = R_2_ = H), H_2_ **L2** [R_1_ = R_2_ = C(CH_3_)_3_] and H_2_ **L3** (R_1_ = F, R_2_ = H) and the reaction scheme for the synthesis of compounds **1−3**.

This ligand can be synthesized in one high-yielding condensation reaction from commercial salicylaldehydes and amino alcohols derived from chiral α-amino acids. Both enantiomers of the ligands are therefore readily accessible. This ligand scaffold has been reported within 3d-transition metal-based PCs displaying interesting magnetic properties [17–23] but, to the best of our knowledge, has never been used in 3d/4f chemistry to yield well-defined PCs with application in catalysis. The assembly of H_2_L^R/S^ with nitrate or chloride Co^II^/Y^III^ salts in CH_3_CN or EtOH harvests, in one step, a tetranuclear C_3_-symmetry propeller-shaped entity formulated {[Co^III^_3_Y^III^(**L1**)_6_] (solvent)} (**1** solvent) (see electronic supplementary material).

Derivatives of **1** are diamagnetic, which permits reaction monitoring by both ^1^H and ^89^Y NMR spectroscopy. As such, differences in the coordination environment of the Y centre may be observed. Critically, enantiomeric ligands yielded isoskeletal and enantiomeric PCs, as shown in figure 2. The best yield was obtained when starting with the metal chloride salts in EtOH (79% yield). Crystals were obtained after slow evaporation, which causes the *in situ* oxidation of the Co^II^ salt to the diamagnetic Co^III^ salt. All the materials lose crystallinity upon exposure to air, but with no alteration of their topology (see electronic supplementary material for TGA graphs). These compounds were further characterized by ^1^H, ^15^N, ^89^Y NMR, thermogravimetric analysis (TGA), Circular dichroism (CD), Electrospray ionization mass spectrometry (ESI-MS) and elemental analysis (see electronic supplementary material). Single-crystal X-ray diffraction studies of the isoskeletal [24] species **1^S^-EtOH**, **1^R^-EtOH** and **1^S^-CH_3_CN** were also carried out (electronic supplementary material, table S1). The Flack parameter was close to zero (e.g. −0.026(5) for **1^R^-EtOH**) in each case, allowing confident determination of absolute configuration.

**Figure 2.**
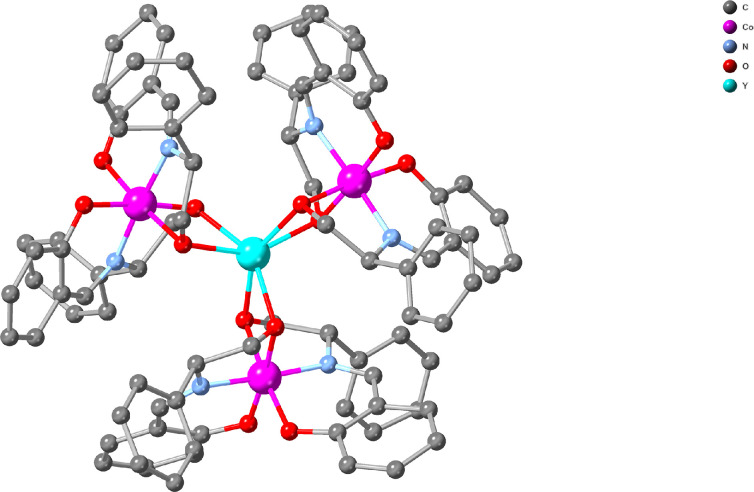
**1^S^-EtOH(100K**); H-atoms and lattice molecules are omitted for clarity. Colour code: Y (cyan), Co (pink), C (light grey), N (light blue), and O (red).

The Y metal sits in the centre with the Co^III^ atoms and associated pairs of organic ligands forming the three propeller wings (figure 2).

Bond distances and angles are within the range that is typical for Co^III^ and Y^III^ centres. The three Co–Y distances span from 3.232(2) to 3.258(2) Å for **1^S^-EtOH**, and in the same range for the other compounds. Two organic ligands are constituents of the octahedral environment of the Co^III^, forming a meridional monoanionic metalloligand {Co^III^(**L1**)_2_}^−^. As noted above, the point chirality within the organic ligand is faithfully translated to the octahedral Co centre. The enantiomerically pure H_2_**L**^**R**^ produces the corresponding meridional {Co^III^(**L1**)_2_}^−^ with Λ-configuration, whereas H_2_**L**^**S**^ yields the Δ-configuration [25,26]. Then three of these metalloligands coordinate to the Y^III^ centre, therefore, its coordination geometry can be best described as distorted trigonal antiprismatic. The {Co^III^(**L1**)_2_}^−^with the Λ-configuration will provide the Λ-enantiomer of the full PC, whereas the {Co^III^(**L1**)_2_}^−^ with the Δ-configuration will give the Δ–enantiomer. A visual representation is provided in electronic supplementary material, figure S1. The dihedral angle of the two planes of the trigonal antiprism (Y^III^ centre) differs (1.15^o^ for **1^R^-EtOH** and 0.73^o^ for **1^S^-EtOH**) and deviates from zero, indicating distortion. This is the only structural difference between these two enantiomers that can be identified. This difference is probably a crystallization artefact and will not persist upon dissolution (cf. figure 3). To obtain better insight into the interaction of the propeller-shaped moiety with lattice solvent molecules, a crystallographic dataset at a lower temperature was recorded: **1^S^-EtOH (100K**). In this case, the oxygen atoms of the salicylic moiety can be seen to participate in H-bonding interactions with the lattice EtOH solvent molecules (electronic supplementary material, figure S3). A space-fill representation shows how the salicylic oxygen atoms point towards the lattice molecules and that the coordination sphere of the Y centre is blocked (electronic supplementary material, figure S4).

**Figure 3.**
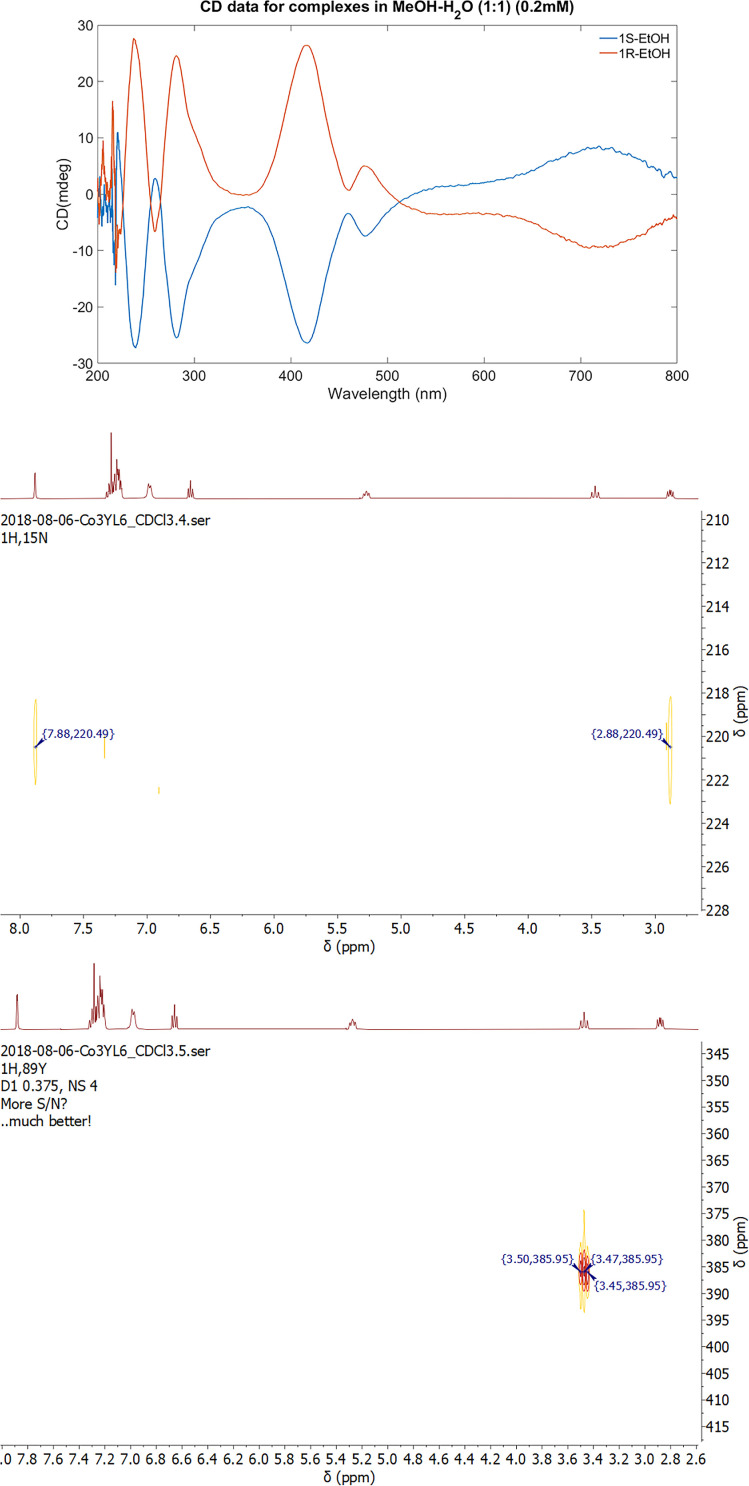
(Upper) circular dichroism data for **1^S^-EtOH** (blue line) and **1^R^-EtOH** (orange line); (middle) ^1^H−^89^Y NMR spectra and (lower) ^1^H–^15^N NMR spectra.

The enantiomeric nature of the PCs in solution was confirmed by CD in MeOH (figure 3). To identify the integrity of the PC in solution, various ESI-MS data in MeOH, EtOH and CH_3_CN as solvents were also recorded. In all cases, a dominant peak of approximately *m*/*z* = 1700 Da was identified, with another minor peak in the region of *m*/*z* = 2200 Da. The latter can be attributed to the formation of a Co^III^_4_Y^III^L_8_ species (electronic supplementary material, scheme S1); however, efforts to isolate this species were unsuccessful, and there was no evidence of this species observed by ^1^H and ^89^Y-NMR spectroscopy even after 24 h. Indeed, its formation may be a by-product of mass spectrometric analysis. The ^1^H and ^89^Y NMR spectra confirm that the solid-state structure determined by X-ray crystallography is retained in the solution state. Coordination of oxygen to the Y centre is confirmed by the observation of ^1^H,^89^Y-HMBC correlations from one of the alkoxy protons at δ_H_ = 3.47 ppm to the ^89^Y centre at δ_Y_ = 386 ppm, mediated by a ^3^J_YH_ = 1.1 Hz coupling that is also observable as a modest broadening of the triplet in the 1D ^1^H spectrum (figure 3). Coordination of Co(III) to the nitrogen centres is also confirmed by the change in ^15^N shift of the free ligand at δ_N_ = 298 ppm to the bound ligand at δ_N_ = 221 ppm*,* i.e. a coordination shift change of −77 ppm [27].

Complexes {[Fe^III^_4_(**L2**)_6_] (solvent)} **2** and {[Co^III^_3_Y^III^(**L3**)_6_] (solvent)} **3** derived from both (*R*)- and (*S*)-ligands have been described previously [22,23]. Building on our previous results [16], we initially investigated the efficacy of **1^S^-EtOH** as a catalyst for the Michael addition reaction of 1,3-dimethyl barbituric acid **4a** and selected derivatives with *trans*-β-nitrostyrene **5**a (table 1). For 1,3-dimethylbarbituric acid **4a**, good yields were obtained after 15 min using 1% catalyst loading in EtOH/H_2_O (3/2) (entries 1−5), but the yields dropped off for the imine derivative **4b** (entries 6−9). Having confirmed the catalytic efficacy of enantiomerically pure PC **1^S^-EtOH**, we decided to examine the ability of this PC and its congeners to induce asymmetry. We selected to switch to chloroform as the solvent for these studies, as it is less polar, and this was anticipated to enhance the influence of non-covalent interactions that typically induce asymmetry in such reactions. We were also interested in the role of the recrystallized solvent molecule that forms part of the unit cell of the PC (MeCN versus EtOH) and the consequent availability of the central Y coordination sphere (table 2).

**Table 1.** The scope of reactions of 1,3-dimethyl barbituric acid **4**a and imine derivative **4b** with *trans*-β-nitrostyrenes **5** catalysed by **1^S^-EtOH**.

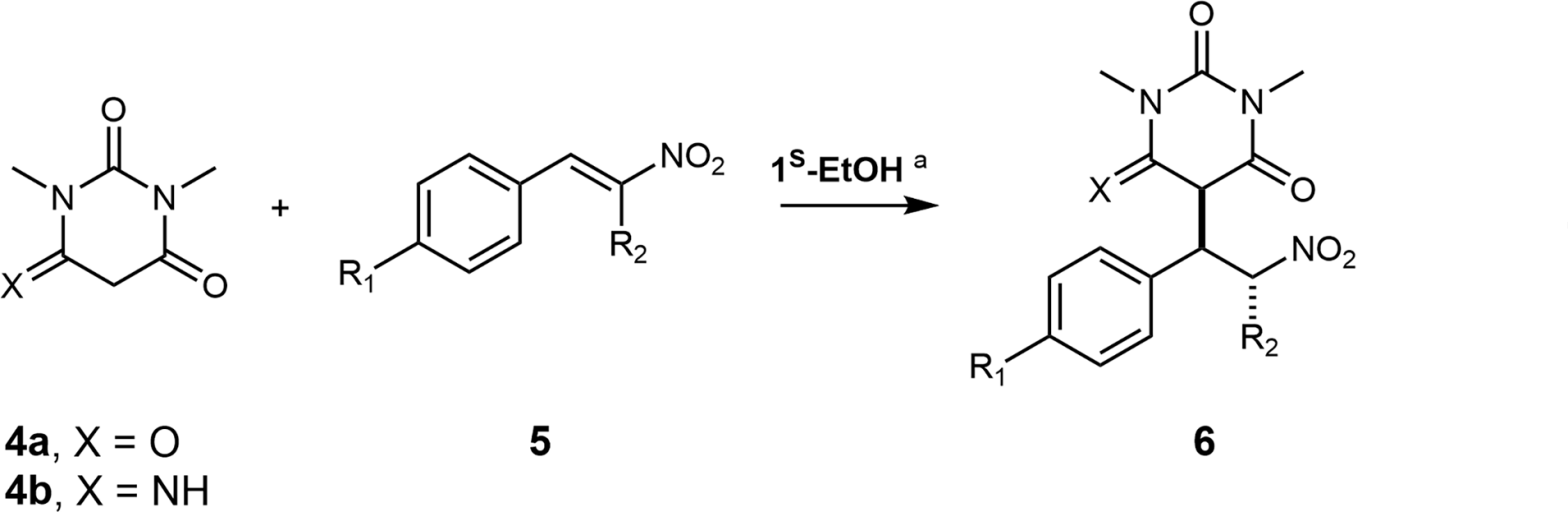				
entry	R_1_	R_2_	X	yield (%)^b^
1	H	H	O	91 (6a)
2	H	CH_3_	O	91^c^(6b)
3	F	H	O	92 (6c)
4	Br	H	O	79 (6d)
5	MeO	H	O	90 (6e)
6	H	H	NH	37 (6f)
7	F	H	NH	48 (6g)
8	Br	H	NH	32 (6h)
9	MeO	H	NH	16 (6i)

^a^ Room temperature, 1,3-dimethylbarbituric acid **4a** (0.25 mmol) *trans*-β nitrostyrene **5** (0.25 mmol, 41 μl), catalyst loading 1%, time 15 mins, solvent EtOH (6 ml), H_2_O (4 ml).

^b^ ^1^H-NMR spectroscopic yield.^16^

^c^ The stereochemistry illustrated for **6** is relative; (1RS,2RS)-diastereoisomer (>20 : 1 dr).

When we used **1^S^-EtOH** as a catalyst, Michael addition product (−)**−6b** was formed with 13% ee (table 2, entry 1). Interestingly, when we used **1^R^-CH_3_CN** as a catalyst, which differs only in the solvent molecule that is crystallized in the unit cell, product **6b** was formed with 0% ee (table 2, entry 2), but, with the addition of EtOH (1.6 equiv.) to the reaction, (+)**−6b** was formed with 7% ee (table 2, entry 3). The use of alternative alcohol additives such as phenols and hexafluoroisopropanol was ineffective in altering the ee (see electronic supplementary material, S12). These experiments suggested that the co-crystallized solvent plays a vital role in stereoinduction. Monitoring the reactions by ^1^H and ^89^Y NMR spectroscopy confirmed that the coordination environment of the Y^III^ centre remains intact. Titration of the complex with 0.7−27 eq. of *trans*-β-methyl-β-nitrostyrene **5b** in CDCl_3_ leads to no change in ^1^H or ^89^Y MR chemical shifts, suggesting that binding of this substrate does not occur. This is in keeping with the X-ray structural data, which show that the Y centre is sterically buried. Collectively, these findings suggest that it is an aspect of the topology of the PC as a whole, or at least substrate–ligand interactions distal from the metal centres rather than those in close proximity to a substrate-interacting metal centre, that are responsible for the, admittedly rather modest, asymmetric induction. To further explore this, complexes **3^R^-CH_3_CN** and **3^S^-CH_3_CN**, which feature fluorine substituents *para* to the phenol group in the chiral ligands (figure 1), were tested as catalysts. These catalysts behaved very similarly, affording the Michael addition products, (+)**−6b** and (−)**−6b**, with a modest ee of 9% and 12%, respectively (table 2, entries 4 and 5).

To test the hypothesis that macromolecular catalyst symmetry could be responsible for enantioselectivity, we decided to explore the catalytic activity of the topologically equivalent, well-defined propeller-shaped PCs, Fe^III^_4_ (**2^R^-Fe_4_-EtOH** and **2^S^-Fe_4_-EtOH**), in this reaction (table 2, entries 6−10). A report has confirmed the integrity and oxidation state of these Fe^III^_4_ centres in the solid state and solution (figure 4 and electronic supplementary material, figures S5 and S6) [22]. The reason for exploring these particular complexes as potentially analogous catalysts was twofold: first, different metal centres may yield different substrate-binding behaviour (i.e. Fe versus Co/Y), and second, the presence of the bulky groups on the salicyl ligands may influence the approach of the substrates to the metal centres (i.e. 2 × C(CH_3_)_3_ versus 2 × H, H/F). Although both **2^R^-Fe_4_-EtOH** and **2^S^-Fe_4_-EtOH** PC complexes efficiently catalysed the Michael addition reaction, the product **6b** was essentially racemic (ee = 0 ± 3) regardless of the presence or absence of EtOH as an additive (table 2, entries 6−10).

**Figure 4.**
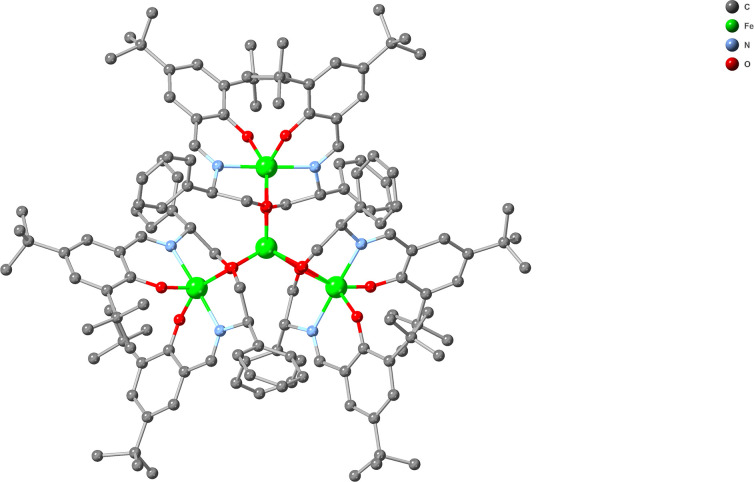
A projection of compound **2^S^-Fe_4_-EtOH**. Colour code: Fe (green), C (light grey), N (light blue) and O (red).

Given that this outcome was in contrast with the modest, but definitively non-zero, enantioselectivities observed for the bimetallic Co/Y complexes **1^S^-EtOH, 1^R^-CH_3_CN, 3^R^-CH_3_CN** and **3^S^-CH_3_CN**, we investigated whether there is leaching of free Co^III^ and/or Y^III^ metal cations from the bimetallic Co/Y cluster complexes. These metal cations could perhaps ligate to the ligands and facilitate the asymmetric Michael addition reaction. We performed control experiments by reacting equimolar quantities of 1,3-dimethyl barbituric acid **4**a with *trans*-β-methyl-β-nitrostyrene **5b** under the standard conditions but with (i) no catalyst or metal salts; (ii) 6 mol% of the ligand **L1** + 3 mol% of Co(NO_3_)_2_ + 1 mol% of Y(NO_3_)_3_ + 1.6 equiv. of EtOH; and (iii) 6 mol% of the ligand **L1** alone with no metal sources. These control reactions were monitored periodically by ^1^H-NMR spectroscopy at *t* = 5 min, 24 h, 48 h and 72 h. No trace of the Michael addition product **6b** was observed during these studies (electronic supplementary material, figure S11). This demonstrates that the reactions are exclusively catalysed by the bimetallic Co/Y cluster complexes and not by the residual metal ions and/or ligands leached from these complexes. The chiral topology of the bimetallic Co/Y cluster complexes appears to directly control the enantioselectivity of this process.

**Table 2.** Enantioselective Michael addition reactions catalysed by propeller-shaped PCs.^a^ Abbreviations: N.D. = not determined; ee = enantiomeric excess; equiv. = equivalents; cat. = catalyst; NCS = N chlorosuccinimde; mol% = mole percentage; h = hour(s)

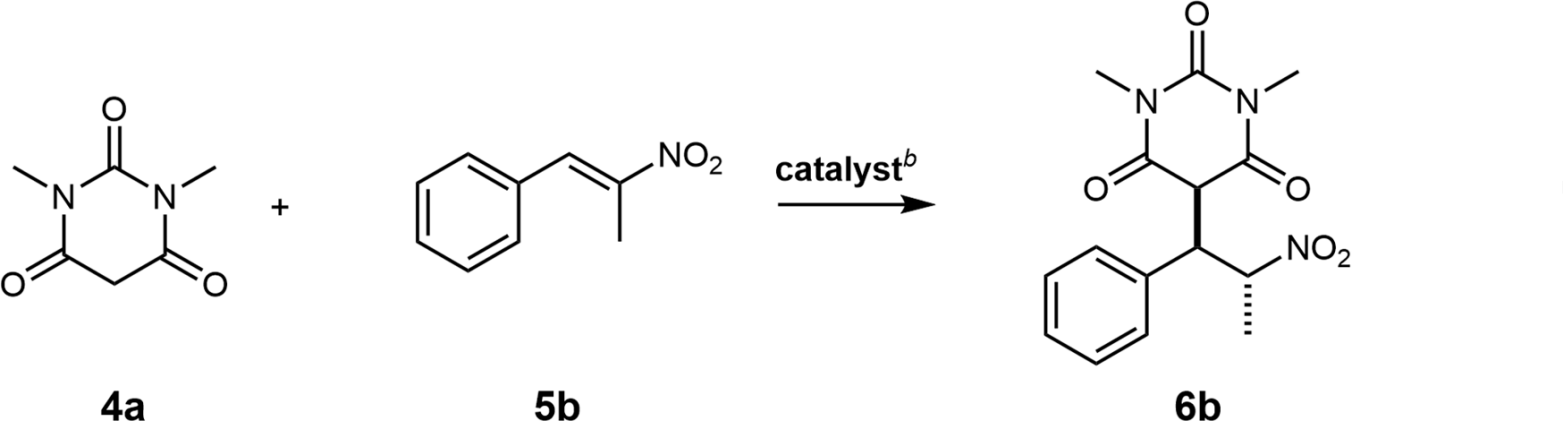					
entry	catalyst^b^	EtOH additive (equiv.)	conversion to 6b (%)^c^	isolated yield of 7b (%)^d^	ee (%)^e^**^,^^f^**^,^^g^ ^,^^h^
1	1^S^-EtOH	—	N.D.	N.D.	13 [(−)−7b]
2	1^R^-CH_3_CN	—	—	—	0
3	1^R^-CH_3_CN	1.6	80	N.D.	7 [(+)−7b]
4	3^R^-CH_3_CN	1.6	64	54	9 [(+)−7b]
5	3^S^-CH_3_CN	1.6	48	41	12 [(−)−7b]
6	2^R^-Fe_4_-EtOH	—	66	38	1 [(−)−7b]
7	2^R^-Fe_4_-EtOH	1.6	67	61	3 [(−)−7b]
8	2^S^-Fe_4_-EtOH	—	75	39	1 [(+)−7b]
9	2^S^-Fe_4_-EtOH	1.6	75	42	2 [(+)−7b]

^a^ For control reactions, see main text and electronic supplementary material, section S14.

^b^ 1,3-dimethylbarbituric acid **4a** (200 μl of 0.1 mM solution in CDCl_3_, 7.81 mg, 0.05 mmol, 1.0 equiv.), *trans*-β-methyl-β-nitrostyrene **5b** (200 μl of 0.1 mM solution in CDCl_3_, 8.16 mg, 0.05 mmol, 1.0 equiv.), CDCl_3_ (200 μl) and catalyst (200 μl of a solution of CDCl_3_ containing 0.0005 mmol, 0.01 eq., 1 mol%). Time 24 h, solvent: CDCl_3_ (800 μl).

^c^ The conversions were determined from ^1^H NMR spectroscopic analysis of the crude reaction mixtures, see electronic supplementary material, section S11.

^d^ Isolated yields were calculated after two consecutive steps from starting materials **4a** and **5b**, after treatment of **6b** with N-chlorosuccinimide (2 equiv.) for 1 h at room temperature and subsequent purification by preparative TLC.

^e^ The absolute configuration of the enantiomers was not determined; the stereochemistry illustrated for **6b** is relative.

^f^ Enantiomeric excess (ee) of the product **6b** was determined by CSP-HPLC analysis of its chlorinated product **7b**.

^g^ The optical rotation of the chlorinated product **7b** was determined by CD spectroscopy (see electronic supplementary material, section S13).

^h^ The ee values were determined by running chiral HPLC analyses for each sample at least twice to ensure consistent and concordant ee values.

In asymmetric catalysis, many cooperative factors may influence the enantioselectivity, including the overall symmetry of the complex [28]. In the present communication, by monitoring the catalyst and performing control experiments with analogously shaped molecules, we have demonstrated that it is possible to slightly influence the stereoselectivity of an organic transformation by retaining the overall topology of propeller-shaped complexes in the absence of metal–substrate binding. This is in contrast to the mechanisms by which bimetallic catalysts have previously been shown to activate reactants via direct cooperative interactions with the dual-metal centres [16] or via metallic interactions on the surface of PCs [29]. Notably, the observed sense of enantiomeric induction in the reactions studied depends on the overall topology of the catalyst. A recent example has demonstrated propellers that can sense the chirality of various organic solvents [30]. However, to the best of our knowledge, this is the first example of asymmetric induction attributable to the chiral propeller topology of 3d/4f PCs. Further research designed to aid our understanding of how the surface interactions of these PCs lead to reaction rate enhancement and what molecular mechanisms are operative leading to enantiomeric enrichment in the products of this type of reaction is underway in our laboratories. We consider the present metal-based catalyst as a new type of catalyst, which offers a conceptually new approach to asymmetric catalysis by coordination chemistry of 3d/4f-based materials.

## Data Availability

Crystal structures have been deposited in Cambridge Structural Database (CSD)—managed by the Cambridge Crystallographic Data Centre (CCDC)—and have been given CCDC deposition numbers 2172015, 2172016, 2172017 Supplementary material is available online [31].

## References

[1] Kanady JS, Tsui EY, Day MW, Agapie T. 2011 A synthetic model of the Mn_3_Ca subsite of the oxygen-evolving complex in photosystem II. Science **333**, 733–736. (10.1126/science.1206036) 21817047

[2] Han Z, Horak KT, Lee HB, Agapie T. 2017 Tetranuclear manganese models of the OEC displaying hydrogen bonding interactions: application to electrocatalytic water oxidation to hydrogen peroxide. J. Am. Chem. Soc. **139**, 9108–9111. (10.1021/jacs.7b03044) 28587453 PMC5643074

[3] Okamura M *et al*. 2016 A pentanuclear iron catalyst designed for water oxidation. Nature New Biol. **530**, 465–468. (10.1038/nature16529) 26863188

[4] Evangelisti F, Moré R, Hodel F, Luber S, Patzke GR. 2015 3d-4f {Co(II)_3_Ln(OR)_4_} cubanes as bio-inspired water oxidation catalysts. J. Am. Chem. Soc. **137**, 11076–11084. (10.1021/jacs.5b05831) 26266575

[5] Handa S, Gnanadesikan V, Matsunaga S, Shibasaki M. 2007 syn-Selective catalytic asymmetric nitro-Mannich reactions using a heterobimetallic Cu−Sm−Schiff base complex. J. Am. Chem. Soc. **129**, 4900–4901. (10.1021/ja0701560) 17394322

[6] Matsunaga S, Shibasaki M. 2014 Recent advances in cooperative bimetallic asymmetric catalysis: dinuclear Schiff base complexes. Chem. Commun **50**, 1044–1057. (10.1039/C3CC47587E) 24281133

[7] Clement ML, Grice KA, Luedtke AT, Kaminsky W, Goldberg KI. 2014 Platinum(II) olefin hydroarylation catalysts: tuning selectivity for the anti-Markovnikov product. Chemistry **20**, 17287–17291. (10.1002/chem.201405174) 25377546

[8] Shul’pin GB. 2013 C-H functionalization: thoroughly tuning ligands at a metal ion, a chemist can greatly enhance catalyst’s activity and selectivity. Dalton Trans. **42**, 12794–12818. (10.1039/c3dt51004b) 23873447

[9] Harada K, Sekiya R, Haino T. 2024 Kinetic resolution of secondary alcohols catalyzed at the exterior of chiral coordinated capsules. Eur. J **30**, e202304244. (10.1002/chem.202304244) 38240735

[10] Hong SY, Park Y, Hwang Y, Kim YB, Baik MH, Chang S. 2018 Selective formation of γ-lactams via C-H amidation enabled by tailored iridium catalysts. Science **359**, 1016–1021. (10.1126/science.aap7503) 29496875

[11] Pye DR, Mankad NP. 2017 Bimetallic catalysis for C–C and C–X coupling reactions. Chem. Sci. **8**, 1705–1718. (10.1039/C6SC05556G) 29780450 PMC5933431

[12] Sammis GM, Danjo H, Jacobsen EN. 2004 Cooperative dual catalysis: application to the highly enantioselective conjugate cyanation of unsaturated imides. J. Am. Chem. Soc. **126**, 9928–9929. (10.1021/ja046653n) 15303860

[13] Peng JB, Kong XJ, Zhang QC, Orendáč M, Prokleška J, Ren YP, Long LS, Zheng Z, Zheng LS. 2014 Beauty, symmetry, and magnetocaloric effect—four-shell keplerates with 104 lanthanide atoms. J. Am. Chem. Soc. **136**, 17938–17941. (10.1021/ja5107749) 25495563

[14] Griffiths K, Kumar P, Akien GR, Chilton NF, Abdul-Sada A, Tizzard GJ, Coles SJ, Kostakis GE. 2016 Tetranuclear Zn/4f coordination clusters as highly efficient catalysts for Friedel–Crafts alkylation. Chem. Commun. **52**, 7866–7869. (10.1039/C6CC03608B) 27248829

[15] Kuang GC, Guha PM, Brotherton WS, Simmons JT, Stankee LA, Nguyen BT, Clark RJ, Zhu L. 2011 Experimental investigation on the mechanism of chelation-assisted, copper(II) acetate-accelerated azide–alkyne cycloaddition. J. Am. Chem. Soc. **133**, 13984–14001. (10.1021/ja203733q) 21809811 PMC3164943

[16] Griffiths K, Tsipis AC, Kumar P, Townrow OPE, Abdul-Sada A, Akien GR, Baldansuren A, Spivey AC, Kostakis GE. 2017 3D/4F coordination clusters as cooperative catalysts for highly diastereoselective Michael addition reactions. Inorg. Chem. **56**, 9563–9573. (10.1021/acs.inorgchem.7b01011) 28783350

[17] Zhu YY, Zhang YQ, Yin TT, Gao C, Wang BW, Gao S. 2015 A family of Co(II)Co(III)_3_ single-ion magnets with zero-field slow magnetic relaxation: fine tuning of energy barrier by remote substituent and counter cation. Inorg. Chem. **54**, 5475–5486. (10.1021/acs.inorgchem.5b00526) 25984913

[18] Mayans J, Font-Bardia M, Bari L, Górecki M, Escuer A. 2018 Chiral [Mn^II^Mn^III^_3_M′] (M′=Na^I^, Ca^II^, Mn^II^) and [Mn^II^Mn^III^_6_Na^I^_2_] clusters built from an enantiomerically pure Schiff base: synthetic, chiroptical, and magnetic properties. Chem. Eur. J. **24**, 18705–18717. (10.1002/chem.201803730) 30230054

[19] Pilichos E, Escuer A, Font-Bardia M, Mayans J. 2020 Chiral versus non-chiral [Mn^III^_6_Mn^II^Na^I^], [Mn^III^_6_Mn^II^_2_Na^I^_2_] and [Mn^III^_3_Mn^II^Na^I^] clusters derived from Schiff bases or the fight for symmetry. Chem. Eur. J. **26**, 13053–13062. (10.1002/chem.202001656) 32428307

[20] Escuer A, Mayans J, Font-Bardia M, Górecki M, Bari L. 2017 Syntheses, structures, and chiroptical and magnetic properties of chiral clusters built from Schiff bases: a novel [Mn^II^MnNa] core. Dalton Trans. **46**, 6514–6517. (10.1039/c7dt00811b) 28426054

[21] Hu P, Wang XN, Jiang CG, Yu F, Li B, Zhuang GL, Zhang T. 2018 Nanosized chiral [Mn_6_Ln_2_] clusters modeled by enantiomeric Schiff base derivatives: synthesis, crystal structures, and magnetic properties. Inorg. Chem. **57**, 8639–8645. (10.1021/acs.inorgchem.8b01423) 29962201

[22] Mayans J, Font-Bardia M, Escuer A. 2018 Chiroptical and magnetic properties of star-shaped Fe complexes from chiral Schiff bases: structural and magnetic correlations based on continuous shape measures. Dalton Trans. **47**, 8392–8401. (10.1039/c8dt01684d) 29897079

[23] Audsley G *et al*. 2023 Chiral Co_3_Y propeller-shaped chemosensory platforms based on ^19^F-NMR. Inorg. Chem. **62**, 2680–2693. (10.1021/acs.inorgchem.2c03737) 36716401 PMC9930122

[24] Griffiths K, Dokorou VN, Spencer J, Abdul-Sada A, Vargas A, Kostakis GE. 2016 Isoskeletal Schiff base polynuclear coordination clusters: synthetic and theoretical aspects. CrystEngComm **18**, 704–713. (10.1039/C5CE02109J)

[25] Legg JI, Douglas BE. 1966 A general method for relating the absolute configurations of octahedral chelate complexes. J. Am. Chem. Soc **88**, 2697–2699. (10.1021/ja00964a013)

[26] Belokon YN, Maleev VI, North M, Larionov VA, Savel’yeva TF, Nijland A, Nelyubina YV. 2013 Chiral octahedral complexes of Co^III^ as a family of asymmetric catalysts operating under phase transfer conditions. ACS Catal. **3**, 1951–1955. (10.1021/cs400409d)

[27] Pazderski L. 2013 ^15^N and ^31^P NMR coordination shifts in transition metal complexes with nitrogen- and phosphorus-containing heterocycles. Annu. Reports NMR Spectrosc. **80**, 33–179. (10.1016/B978-0-12-408097-3.00002-0)

[28] Moberg C. 1998 C_3_ symmetry in asymmetric catalysis and chiral recognition. Angew. Chemie Int. Ed. **37**, 248–268. (10.1002/(sici)1521-3773(19980216)37:3<248::aid-anie248>3.0.co;2-5) 29711253

[29] Cruchter T, Larionov VA. 2018 Asymmetric catalysis with octahedral stereogenic-at-metal complexes featuring chiral ligands. Coord. Chem. Rev. **376**, 95–113. (10.1016/j.ccr.2018.08.002)

[30] Shimizu Y, Shoji Y, Hashizume D, Nagata Y, Fukushima T. 2018 Sensing the chirality of various organic solvents by helically arranged π-blades. Chem. Commun **54**, 12314–12317. (10.1039/C8CC06277C) 30221285

[31] Maddocks J, Mahesh M, Sampani SI, Dixon AC, Nielsen CDT, Kumar P *et al*. 2025 Supplementary material from: Single Enantiomer Propeller-shaped Polynuclear Complexes as Catalysts – Proof-of-Concept for Enantioselective Induction in a Michael Addition Reaction. Figshare. (10.6084/m9.figshare.c.7734466) PMC11947762 40151485

[32] Coles SJ, Gale PA. 2012 Changing and challenging times for service crystallography. Chem. Sci **3**, 683–689. (10.1039/C2SC00955B)

